# Synergistic Effects of Acetic Acid and Ethanol on Offspring Production and Gallery Expansion by Fungus-Farming Ambrosia Beetles

**DOI:** 10.1007/s10886-026-01725-3

**Published:** 2026-05-30

**Authors:** Christopher M. Ranger, Julie A. Baniszewski, Nisha Patwa, Hsin-Ho Wei, Sudhan Pachhain, Jessi A. Raubenolt, Madeline Altland, Juan Carlos Cambronero-Heinrichs, Vipaporn Phuntumart, Michael E. Reding, Davide Rassati, Ian W. Keesey, Peter H. W. Biedermann

**Affiliations:** 1 Horticultural Insects Research Laboratory, U.S. Department of Agriculture–Agricultural Research Service, Wooster, OH 44691 USA; 2 Department of Entomology, The Ohio State University, Wooster, OH 44691 USA; 3 Department of Entomology, Hampton Roads Agricultural Research and Extension Center, Virginia Polytechnic Institute and State University, Virginia Beach, VA 23455 USA; 4 Department of Biological Sciences, Bowling Green State University, Bowling Green, OH 43403 USA; 5 Department of Agronomy, Food, Natural Resources, Animals and Environment (DAFNAE), University of Padova, Viale dell’Università, Legnaro, Padova, 35020 Italy; 6 Programa de Investigación en Enfermedades Tropicales (PIET), Escuela de Medicina Veterinaria, Universidad Nacional de Costa Rica, Heredia, 86-3000 Costa Rica; 7 Centro Nacional de Innovaciones Biotecnológicas (CENIBiot), CeNAT- CONARE, San José, 1174-1200 Costa Rica; 8 School of Biological Sciences, University of Nebraska-Lincoln, Manter Hall 402, Lincoln, NE 68588 USA; 9 Forest Entomology and Forest Protection, Albert Ludwig University of Freiburg, Stegen, 79252 Germany; 10 Future Forests Cluster of Excellence, University of Freiburg, 79014 Freiburg im Breisgau, Germany

**Keywords:** Fungus-farming insects, X-ray micro-computed tomography, Insect–fungus mutualism, Symbiosis

## Abstract

Wood-boring ambrosia beetles and their offspring obligately depend on cultivated fungi that they maintain within host trees. Stressed trees produce at least two potent antimicrobials, ethanol and acetic acid, which typically suppress the growth of most fungi. Ethanol facilitates the growth of ambrosia beetles’ fungal mutualists and thus aids in host colonization; however, acetic acid’s effects are unknown. Here, we evaluated the effects of acetic acid on tunneling and offspring production by two exotic ambrosia beetle species, *Xylosandrus germanus* and *Anisandrus maiche*. Cut wood stems (i.e., bolts) were infused with water alone, 5% ethanol, or mixtures of 5% ethanol with acetic acid at various dilutions. Infestations by *X. germanus* and *A. maiche* resulted in more frass, larvae, pupae, and adults in the combined 5% ethanol-acetic acid treatment, than in the 5% ethanol alone or water control treatments. X-ray micro-computed tomography revealed that bolts infused with this 5% ethanol-acetic acid mixture had larger insect gallery volumes than those with only 5% ethanol, which in turn were larger than the water control. A positive relationship was found between frass ejected by each beetle and gallery volume across all experiments. This study demonstrates that acetic acid and ethanol synergistically increase offspring production while facilitating gallery expansion by the larvae. Understanding how ambrosia beetles utilize stress-induced compounds within host trees to benefit their fungiculture could lead to novel management strategies for these invasive insects.

**Supplementary Information:**

The online version contains supplementary material available at 10.1007/s10886-026-01725-3.

## Introduction

Herbivorous insects are exposed to host-derived allomones that are either constitutively present or actively induced when the herbivores begin to feed on plant tissues (Whitehill et al. 2023). Specialized, co-evolved herbivores, however, are typically well adapted to cope with these defenses and can often even make use of the defensive plant chemistry for their own benefits through sequestration or modification into other useful compounds (Whitehill et al. 2023). Some bark beetles (Curculionidae: Scolytinae), for example, degrade the monoterpene defenses of conifer trees to produce aggregation pheromones and even generate anti-aggregation pheromones, which they use for their interspecific communication during host tree colonization (Frühbrodt et al. 2024). Interestingly, they may also use these same monoterpene substances as kairomones to find their preferred host tree species (Miller and Rabaglia 2009).

Ambrosia beetles represent a group of highly-derived bark beetles that are characterized by their obligate nutritional relationship with symbiotic fungi (Hulcr and Stelinski 2017). While some destructive species of invasive ambrosia beetles select healthy trees (Martini et al. 2015; Hulcr and Stelinski 2017), other key species preferentially infest trees that are physiologically weakened, particularly those experiencing flood or freeze stress (LaSpina et al. 2013; Ranger et al. 2021; Cambronero-Heinrichs et al. 2024, 2025). When plants and trees are exposed to anoxic or hypoxic conditions, they produce and release higher amounts of by-products from fermentative metabolism than healthy ones, including increases in ethanol, acetaldehyde, and acetic acid (Kimmerer and Kozlowski 1982; Kimmerer and MacDonald 1987; Vartapetian and Jackson 1997; Rottenberger et al. 2008; Jardine and McDowell 2023). Pyruvate decarboxylase and alcohol dehydrogenase result in the production of acetaldehyde and ethanol, and aldehyde dehydrogenase is believed to further oxidize most acetaldehyde to acetic acid (Kimmerer and Kozloweski 1982, Wei et al. 2009; Kreuzwieser and Rennenberg 2014).

Highly destructive ambrosia beetles that infest healthy trees, such as *Xyleborus glabratus* Eichhoff and *Euwallacea fornicatus* Eichhoff, are weakly or not attracted to ethanol (Martini et al. 2015; Hulcr and Stelinski 2017). Conversely, host-derived ethanol serves as a long-range attractant for some invasive ambrosia beetles that preferentially colonize stressed trees, such as *Xylosandrus germanus* (Blandford), *Xylosandrus crassiusculus* (Motschulsky), and *Anisandrus maiche* (Kurentsov) (Ranger et al. 2021; Yilmaz et al. 2024). In response to the presence of ethanol, adult female ambrosia beetles tunnel into the sapwood and heartwood of trees to create galleries where they cultivate fungal mutualists and raise their offspring (Ranger et al. 2015, 2018; Rassati et al. 2020; Cavaletto et al. 2023). For the ethanol-responsive ambrosia beetle *X. germanus*, ethanol promotes the growth of its fungal mutualist, *Ambrosiella grosmanniae* Mayers, McNew, & Harr., thereby enhancing tree colonization by the foundresses. In turn, ethanol suppresses the growth of ‘weedy’ fungi that compete with this nutritional symbiont (Ranger et al. 2018; Lehenberger et al. 2021a, b).

A growing body of evidence suggests that another antimicrobial agent, acetic acid, may also serve as a key semiochemical that aids some ambrosia beetles in locating and colonizing stressed host trees. Acetic acid was detected in emissions from beech, *Fagus sylvatica* L., wood colonized by *X. germanus*, but not uncolonized wood (Wehnert 2014). It was also detected in emissions from flood-stressed *Cornus florida* L. infested by *X. germanus* but not in non-flooded trees (Ranger et al. 2013). A combination of acetic acid and ethanol lures increased trap captures of *X. germanus* by 206% compared to ethanol alone (Wehnert 2014), which suggests acetic acid represents an olfactory cue during host finding. Due to its insecticidal and fungicidal properties (Tiilikkala et al. 2010), hardwood vinegar was initially tested as a treatment against ambrosia beetles (Viloria et al. 2019). Unexpectedly, three times more *X. crassiusculus* emerged from *Malus domestica* bolts that were infused with 95% ethanol and treated with hardwood vinegar (which contains 5% acetic acid) compared to bolts treated with only ethanol alone (Viloria et al. 2019). Acetic acid is the primary component of hardwood vinegar, but numerous other chemical components are also present (Theapparat et al. 2018; Mengfan et al. 2022). Additionally, bolts from *Acer rubrum* L., *Acer platanoides* L., and *M. domestica* were more attractive to *X. crassiusculus* and *X. germanus* when soaked in mixtures of acetic acid and ethanol compared to those soaked in acetic acid alone or ethanol alone (Reding et al. 2025). These bolts also produced more offspring than those treated with either acetic acid or ethanol alone (Reding et al. 2025).

Fungus-farming insects depend on a carefully regulated feedback loop (i.e., mutualism management) between the farmers and their fungal crops for success (Mueller et al. 2005, Biedermann and Rohlfs 2017). Pheromones have been shown to coordinate a wide range of social activities in ant and termite societies (Hölldobler et al. 1990, Zhou et al. 2026; Mitaka and Akino 2021). Similarly, their substrate’s chemical profile influences the behavior and cooperation of fungus-farming ants and termites, including avoidance learning (Arenas and Roces 2017; Goes et al. 2020), sanitary and hygienic behaviors (Kyle et al. 2023), and social coordination (Ocko et al. 2019). The influence of host tree chemistry, particularly ethanol and acetic acid, on social activities by the fungus-farming ambrosia beetles is less well understood.

For fungus-farming to succeed, cooperation between adults and offspring can occur to support population growth (Biedermann and Taborsky 2011; Schultz and Brady 2008). Unlike the obligate eusociality found in farming ants and termites, agriculture in ambrosia beetles is facultative, with individuals exhibiting plasticity between solitary and social behaviors (Biedermann and Taborsky 2011; Biedermann 2020). Notably, ambrosia beetle foundresses shift from initial gallery construction and tending to the fungus to primarily blocking the gallery entrance to prevent the entry of predators and parasitoids, regulating gallery humidity, and containing larvae when brood is present (Biedermann and Taborsky 2011; Nuotclà et al. 2014; Melet et al. 2024; Milbrath et al. 2024). Research on *Xyleborinus saxesenii* (Ratzeburg) indicates that adult and larval ambrosia beetles occupy different feeding guilds; adult females produce frass with an enzymatic profile distinct from that of larval frass, suggesting social-structure differences and behavioral cooperation (De Fine Licht and Biedermann 2012). The larvae also consume fungus-infested wood while feeding on the fungal gardens (De Fine Licht and Biedermann 2012), thereby contributing to gallery expansion. This xylo-mycetophagous feeding habit of larvae is typical for species in the genera *Anisandrus*,* Xyleborinus* and *Xylosandrus* (Roeper 1995). While studies have shown that larvae can facilitate this expansion in artificial media (Biedermann and Taborsky 2011; Milbrath et al. 2024), it remains unclear whether host-plant metabolites stimulate gallery expansion.

**Fig. 1:**
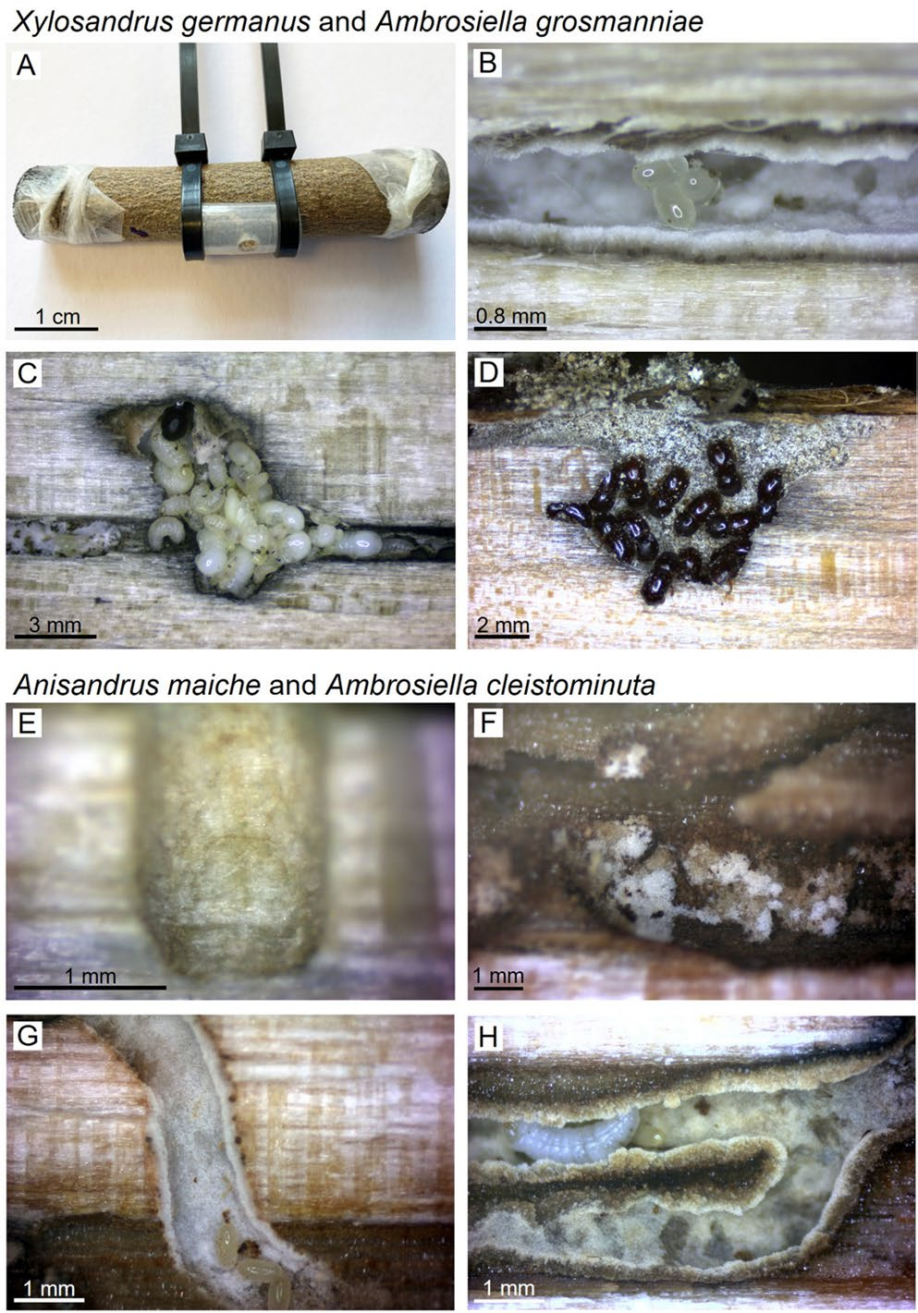
**A-H**.** A** Stem section (i.e., bolt) of *Cornus florida* infused with water or dilutions of acetic acid (AcOH) and/or ethanol (EtOH) with a no-choice chamber to confine *Xylosandrus germanus* (shown) and *Anisandrus maiche* (not shown) to bolts. Ejected frass is present within the no-choice chamber. **B** The fungal mutualist *Ambrosiella grosmanniae* is shown with *X. germanus* eggs, (**C**) larvae, pupae, and (**D**) adults within *C. florida* bolts infused with a mixture of 5% ethanol and 5% acetic acid. Galleries of *A. maiche* and *Ambrosiella cleistominuta* within bolts of *Acer saccharum* infused with (**E**) water, (**F**) 5% ethanol, (**G**) 5% acetic acid, and (**H**) 5% ethanol plus 5% acetic acid

Our study aimed to assess the influence of acetic acid and ethanol on the colonization of wood by two ethanol-responsive species of ambrosia beetles, *X. germanus* and *A. maiche*. Both species are invasive in eastern North America and infest trees in managed and natural systems (Ranger et al. 2016; Tobin et al. 2024). Specifically, female *X. germanus* and *A. maiche* target living but weakened trees in the early stages of physiological stress induced by abiotic factors, such as flooding or freezing (Ranger et al. 2019, 2023; Cambronero-Heinrichs et al. 2024). We conducted a series of no-choice bioassays (Fig. 1A-H) as part of our current study to further understand the role of stress-induced semiochemicals on colonization by *X. germanus* and *A. maiche*. We hypothesized that the combination of these stress-induced metabolites would promote offspring production and stimulate larvae to participate in gallery expansion, thereby increasing farming and food production.

## Materials and Methods

### Insects

We collected live adult females of *X. crassiusculus* and *X. germanus* in a deciduous woodlot at The Ohio State University, Wayne Co., Ohio (40°45’42” N; 81°51’19” W), as previously described (Ranger et al. 2015). We returned the field-collected specimens to the laboratory and stored them in Petri dishes containing moistened filter paper at 3 °C for 2 to 3 days before use in bolt bioassays.

### Bolt Bioassays with *X. germanus*

Three bioassays evaluated the tunneling behavior and offspring production of *X. germanus* in wood bolts infused with various dilutions of acetic acid and ethanol. Bolts (7.6 cm × 1.9 cm; l × diam.) were prepared from the main stems of ~ 3-year-old flowering dogwood (*C. florida*), a highly susceptible host commonly attacked by this species in ornamental nurseries (Ranger et al. 2016). In the first bioassay, bolts were soaked for 24 h in water, 5% ethanol alone, or acetic acid alone at 0.1%, 1%, 5%, or 10% (v/v). After soaking, bolts were air-dried (100 cfm for 1 h) and the ends wrapped with Parafilm^®^. We confined a single female *X. germanus* in a no-choice chamber constructed from translucent PTFE tubing and Molded Thermogreen LB-2 Septa (Supelco, Bellefonte, PA) (Ranger et al. 2015) (Fig. 1A). A 0.5 cm circular window was created in the tubing using an arch punch (No. 149; Osborne & Co., Harrison, NJ, USA). This opening was sealed with a section of clear polypropylene film (1.4 cm × 1.2 cm, l × w) cut from a commercial sheet protector and secured with clear pressure-sensitive adhesive tape. A ~ 0.1 mm hole was created in the window using a No. 1 insect pin to aid in air exchange. Chambers were secured to the bark using cable ties (Fig. 1A). Beetles that did not tunnel into the stem and were visible on the bark within the no-choice chambers after 24 h were replaced to ensure synchronized colonization; this typically accounted for 5–6% of beetles per bioassay. We replicated the treatments 50 times (*n* = 10 bolts per treatment, repeated five times). After 21 days in an incubator at 25 °C, we weighed the mass of ejected frass using an analytical balance with 0.1 mg readability (AE200, Mettler Toledo, Columbus, OH) and then we dissected the bolts under a stereomicroscope to quantify eggs, larvae, and pupae.

A subsequent no-choice bioassay with *X. germanus* was conducted using *C. florida* bolts soaked for 24 h in water, 5% ethanol, or 5% ethanol plus acetic acid at dilutions of 0.1%, 1%, 5%, or 10% (v/v). The bolts were maintained in an incubator at 25 °C for 21 days, after which the ejected frass per bolt was quantified. We also dissected the bolts to quantify the number of eggs, larvae, and pupae per gallery. Treatments were replicated 32 times (*n* = 8 bolts per treatment, repeated four times).

The final no-choice bioassay with *X. germanus* involved *C. florida* bolts being soaked for 24 h in water, 5% ethanol, or 5% ethanol plus acetic acid at dilutions of 0.1%, 1%, 5%, or 10% (v/v). To ensure sufficient time for *X. germanus* offspring to develop into adults, we incubated the bolts at 25 °C for 35 days. Afterward, we quantified the amount of ejected frass and the number of eggs, larvae, pupae, and adults per gallery were quantified. Treatments were replicated 27 times (*n* = 9 bolts per treatment, repeated three times). We used micro-CT to analyze a subset of these bolts (*n* = 9 bolts per treatment), infused with water, 5% ethanol, or 5% ethanol plus 5% acetic acid. Scanning was conducted 21 days after infestation with *X. germanus*, as described below, and then we returned them to the incubator for the remaining 14 days to determine the impact of gallery expansion on adult emergence.

### Bolt Bioassays with *A. maiche*

To validate the synergistic effect across taxa, bioassays were conducted using *A. maiche* on ~ 3-year-old sugar maple (*Acer saccharum* L.; 2.5 cm diam.) bolts. The genus *Acer* is a well-documented host for this species (Rabaglia et al. 2009), and flood-stressed *A. saccharum* have consistently been observed as a susceptible host under field conditions in Ohio, USA (Ranger, pers. obs). Bolts were soaked for 24 h in water, 5% ethanol, or 5% ethanol plus acetic acid at dilutions of 0.1%, 1%, 2.5%, 5%, or 10% (v/v). Infested bolts were incubated at 25 °C for 35 days to allow offspring to mature into adults. We quantified ejected frass and the number of eggs, larvae, pupae, and adults per gallery. Treatments were replicated 36 times (*n* = 9 bolts per treatment, repeated four times). Following the protocol for *X. germanus*, we used micro-CT scans to analyze a subset of bolts (*n* = 8 bolts per treatment) at 21 days after infestation with *A. maiche*. These bolts were infused with water, 5% ethanol, or 5% ethanol plus 5% acetic acid. After scanning, we returned the bolts to the incubator for the remaining 14 days.

### Ambrosia Beetle Gallery Structure

We used non-destructive micro-CT, a non-destructive method that does not impact insect development or survival (Keszthelyi et al. 2020; Lehmann et al. 2021), to characterize the 3D gallery architecture of *X. germanus* and *A. maiche*. To ensure imaging of well-established galleries, we selected bolts with the highest frass ejection from each treatment (water, 5% ethanol, and 5% ethanol plus 5% acetic acid) in the previously described 35-day bioassays (*n* = 9 for *X. germanus*; *n* = 8 for *A. maiche*). At 21 days post-infestation, bolts were scanned using a Siemens Inveon MicroCT system (Siemens Medical Solutions USA; Cary, NC). Samples were secured horizontally to a carbon fiber sled and underwent a 360° scan (180 rotation steps) at 80 kV and 500 µA. Acquisition lasted about 30 min per bolt, and all samples were scanned and returned to the incubator within a 24-hr period to minimize disruption to beetle development. Exposure time was 160 msec, yielding a spatial resolution of 97 μm. Images were reconstructed using Siemens proprietary software. We segmented and quantified gallery volumes using Amira™ 3D Visualization & Analysis Software v.2024.1 (Thermo Fisher Scientific; Waltham, MA). After scanning, we returned the bolts to the incubator to complete the 35-day development period.

### Molecular Quantification of *A. grosmanniae* Titer

We determined the relative abundance of *A. grosmanniae* within galleries created in *C. florida* bolts infused with water, 5% ethanol, or acetic acid at 0.1%, 1%, 5%, and 10% (v/v) (see the *SI* *Text* for specific methods) (Mayers et al. 2015).

### Acetic Acid and Growth of *A. grosmanniae*

We evaluated the impact of acetic acid on fungal biomass of *A. grosmanniae* OH11 (see the *SI* *Text* for specific methods) (Ranger et al. 2018).

### Quantification of Acetic Acid and Ethanol

Solid-phase microextraction-gas chromatography-mass spectrometry (SPME-GC-MS) was used to measure acetic acid and ethanol concentrations in infused *C. florida* bolts and to compare them with those of experimentally flood-stressed trees, confirming that biologically relevant concentrations were tested in our study (Table S1) (see the *SI* *Text* for specific methods) (Romeo 2009).

### Statistical Analysis

Continuous, non-normal data (e.g., amount of ejected frass, gallery volume, fungal growth, and metabolite concentrations) were analyzed using a generalized linear model (GLM) with a Gamma distribution and log link function. Discrete, non-normal count data (e.g., eggs, larvae, pupae, and adults) were analyzed using a negative binomial distribution and log link function. Model fit was confirmed by ensuring the scaled deviance (*G*^*2*^*/df*) ratio was near 1.0. Mean separations were performed using Tukey’s HSD test (*α* = 0.05) on the least squares means. To evaluate the relationship between ejected frass, as a proxy for excavation activity, and the resulting gallery volume, we performed a Spearman rank-order correlation. All statistical analyses were performed using SAS software, version 9.4 (SAS Institute, Cary, NC).

## Results

### Ethanol-Acetic Acid Amplifies *X. germanus* Offspring Production

**Fig. 2:**
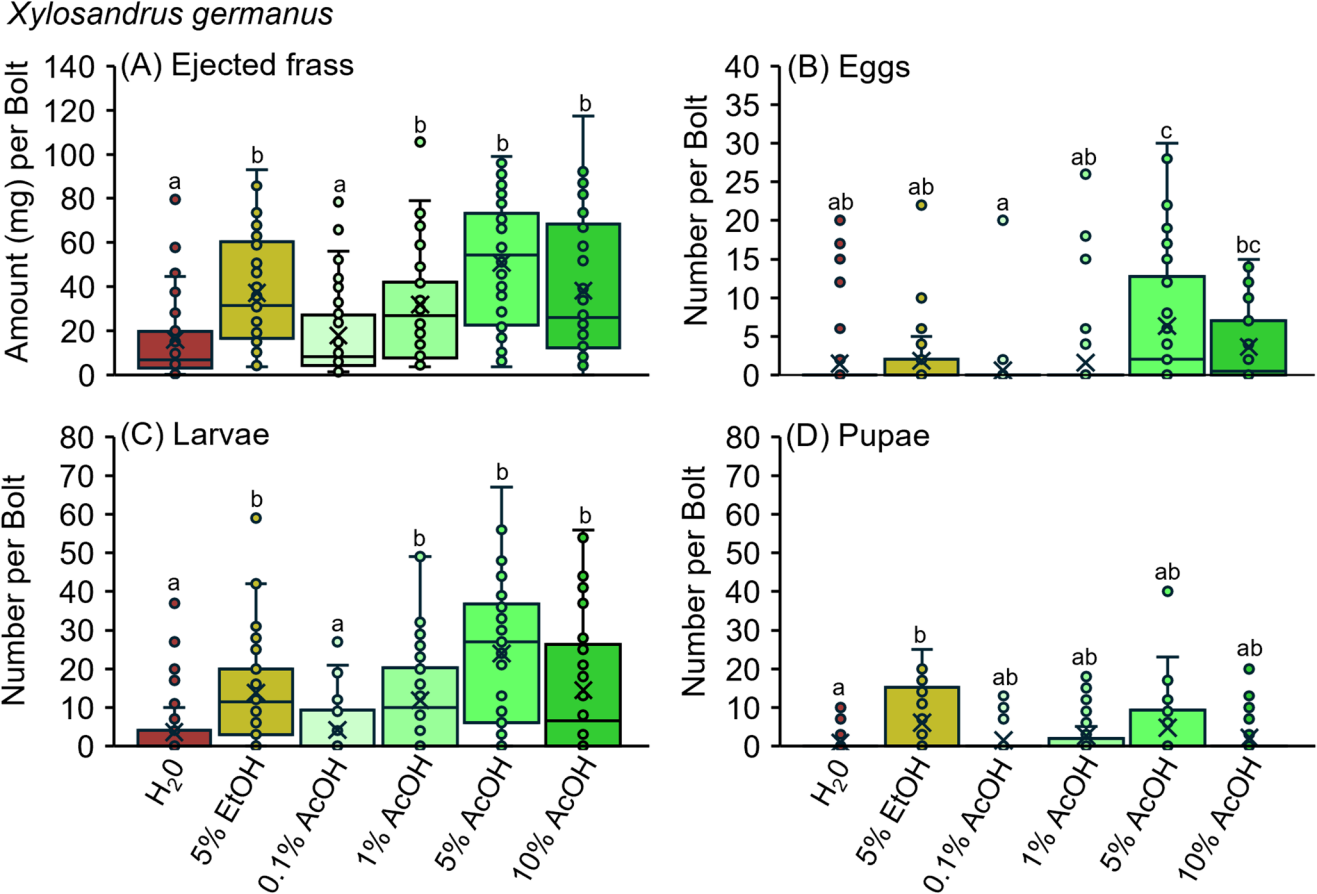
**A-D**. Bolts of *C. florida* infused with dilutions of ethanol (EtOH) and acetic acid (AcOH) were infested with adult *X. germanus* for 21 days, then dissected and quantified for (**A**) ejected frass, (**B**) eggs, (**C**) larvae, and (**D**) pupae. Different letters above bars within each figure represent significantly different means (GLM with Tukey’s HSD: **A***Χ*^*2*^ = 57.69; df = 5; *P* < 0.0001; **B***Χ*^*2*^ = 21.61; df = 5; *P* = 0.0006; **C***Χ*^*2*^ = 35.93; df = 5; *P* < 0.0001; **D** *Χ*^*2*^ = 11.66; df = 5; *P* = 0.040; *n* = 50 bolts per treatment)

The chemical state within host tissues increased *X. germanus* offspring production and increased gallery excavation by larvae. An initial bioassay assessed the tunneling activity and offspring production of *X. germanus* when confined to *C. florida* bolts infused with water, 5% ethanol, and acetic acid dilutions of 0.1%, 1%, 5%, and 10% (Figs. 1A-D and 2A-D). At 21 days after infestation, beetles ejected significantly more frass when confined to bolts infused with 1%, 5%, and 10% acetic acid, along with 5% ethanol, compared to the water control and 0.1% acetic acid (Fig. 2A). However, there was no difference in ejected frass between bolts infused with 0.1% acetic acid and the water control. Dissecting the infested bolts revealed that those infused with 5% acetic acid contained significantly more eggs per gallery than those infused with water, 5% ethanol, or either lower concentration of acetic acid (Fig. 2B). Significantly more larvae were recorded from bolts infused with 1%, 5%, or 10% acetic acid and 5% ethanol compared to the water control (Fig. 2C). However, there was no difference in the number of larvae between bolts infused with 0.1% acetic acid and the water control. There was no significant difference in the number of pupae collected from bolts infused with 0.1%, 1%, 5%, or 10% acetic acid compared to the water control (Fig. 2D). However, more pupae were recovered from bolts infused with 5% ethanol than from the water control.

**Fig. 3:**
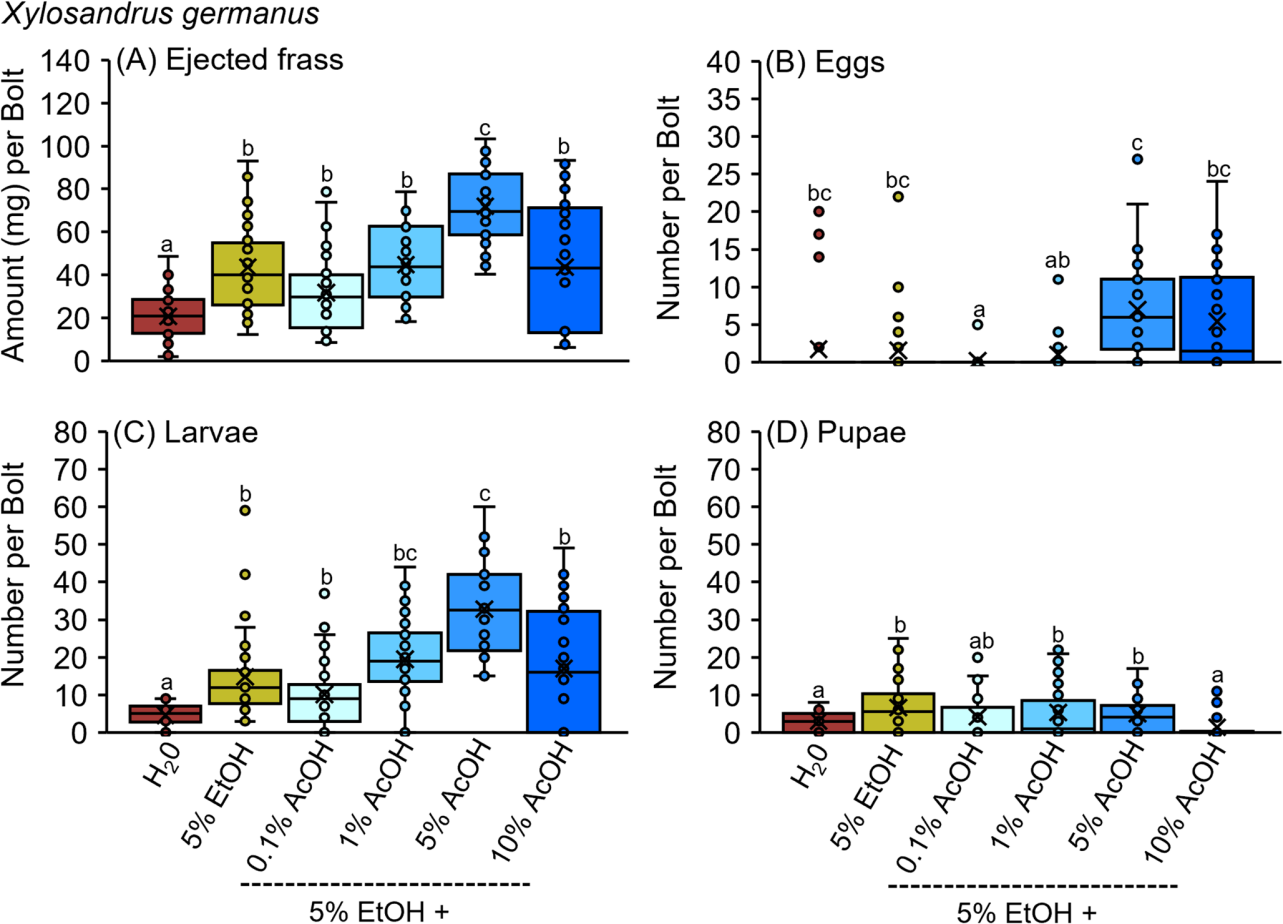
**A-D** Individual female *X. germanus* were confined with no-choice to bolts of *C. florida* infused with dilutions of ethanol (EtOH) and acetic acid (AcOH), which were then dissected after 21 days for (**A**) ejected frass, (**B**) eggs, (**C**) larvae, and (**D**) pupae. Different letters above bars within each figure represent significantly different means (GLM with Tukey’s HSD: **A***Χ*^*2*^ = 64.09; df = 5; *P* < 0.0001; **B***Χ*^*2*^ = 30.02; df = 5; *P* < 0.0001; **C***Χ*^*2*^ = 62.87; df = 5; *P* < 0.0001; **D** *Χ*^*2*^ = 14.80; df = 5; *P* < 0.0001; *n* = 32 bolts per treatment)

A subsequent bioassay evaluated the tunneling activity and offspring production of *X. germanus* confined to *C. florida* bolts infused with water, 5% ethanol, and mixtures of 5% ethanol plus 0.1%, 1%, 5%, and 10% acetic acid (Fig. 3A-D). At 21 days after infesting, beetles ejected significantly more frass from bolts infused with a mixture of 5% ethanol plus 5% acetic acid than from all other treatments (Fig. 3A). There was no difference between 5% ethanol and 5% ethanol plus 0.1%, 5%, and 10% acetic acid. Significantly less frass was ejected from the water control than from any other treatment. No difference was detected in the number of eggs per gallery collected from bolts infused with a mixture of 5% ethanol plus 1%, 5%, or 10% acetic acid compared to 5% ethanol alone or the water control (Fig. 3B). Nevertheless, more larvae were collected from bolts infused with a mixture of 5% ethanol plus 5% acetic acid compared to 5% ethanol alone and the water control (Fig. 3C). Significantly fewer larvae were recorded from the water control than any other treatment. Significantly more pupae were collected from bolts infused with 5% ethanol and mixtures of 5% ethanol plus 1% and 5% acetic acid compared to the water control (Fig. 3D).

**Fig. 4:**
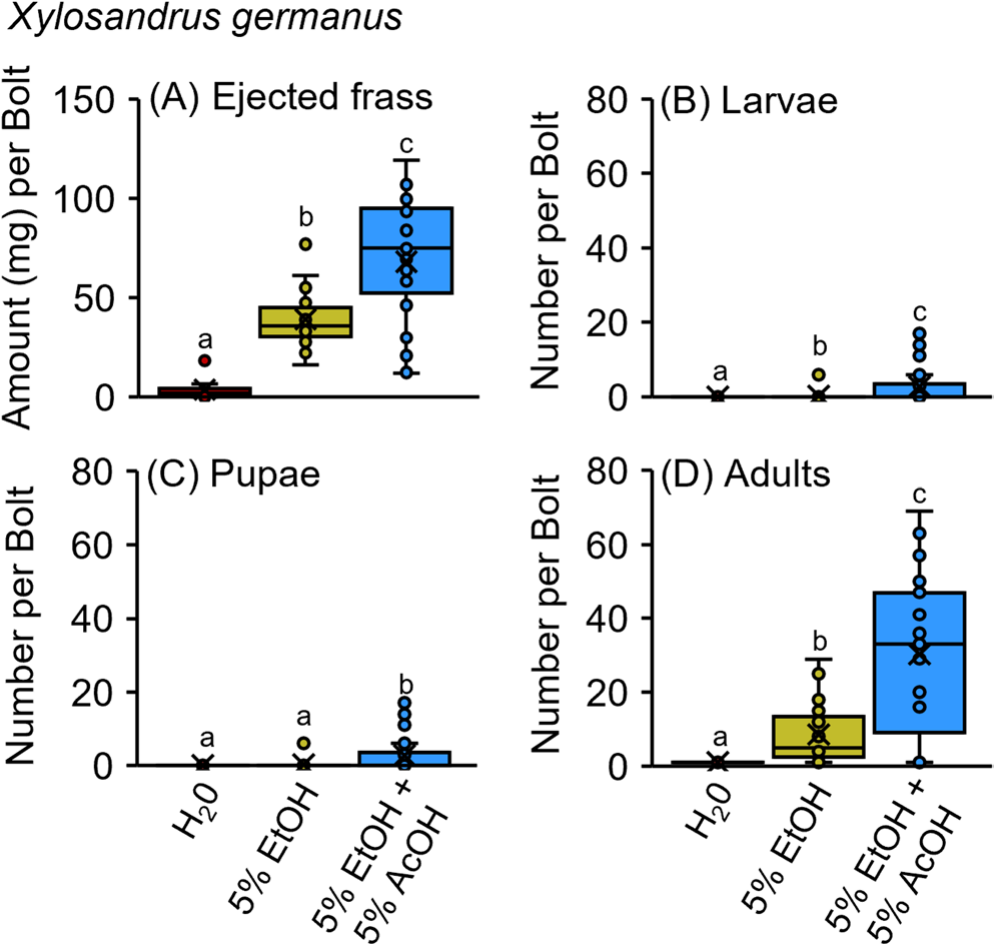
**A-D** Bolts of *C. florida* infused with dilutions of ethanol (EtOH) and acetic acid (AcOH) were infested with adult *X. germanus* for 35 days, then dissected and quantified for (**A**) ejected frass, (**B**) eggs, (**C**) larvae, and (**D**) pupae. Different letters above bars within each figure represent significantly different means (GLM with Tukey’s HSD: **A***Χ*^*2*^ = 95.27; df = 2; *P* < 0.0001; **B***Χ*^*2*^ = 15.90; df = 2; *P* = 0.0004; **C***Χ*^*2*^ = 22.30; df = 2; *P* < 0.0001; **D** *Χ*^*2*^ = 85.19; df = 2; *P* < 0.0001; *n* = 27 bolts per treatment)

In a follow-up bioassay, the infused and infested bolts were held for 35 days instead of 21 days before dissecting to allow the *X. germanus* offspring to mature into adults (Fig. 4A-D). Beetles confined to bolts infused with a mixture of 5% ethanol plus 5% acetic acid ejected significantly more frass than those confined to bolts infused with only 5% ethanol or the water control (Fig. 4A). Significantly more larvae, pupae, and adults were collected from bolts infused with a mixture of 5% ethanol plus 5% acetic acid compared to bolts infused with 5% ethanol alone and the water control (Figs. 4B-D). This treatment produced a 253.7% increase (~ 3.5-fold) in *X. germanus* adults compared to 5% ethanol alone. More ejected frass, larvae, and adults, but not pupae, were associated with bolts infused with 5% ethanol than with the water control.

### Ethanol-acetic Acid Amplifies *A. maiche* Offspring Production

**Fig. 5:**
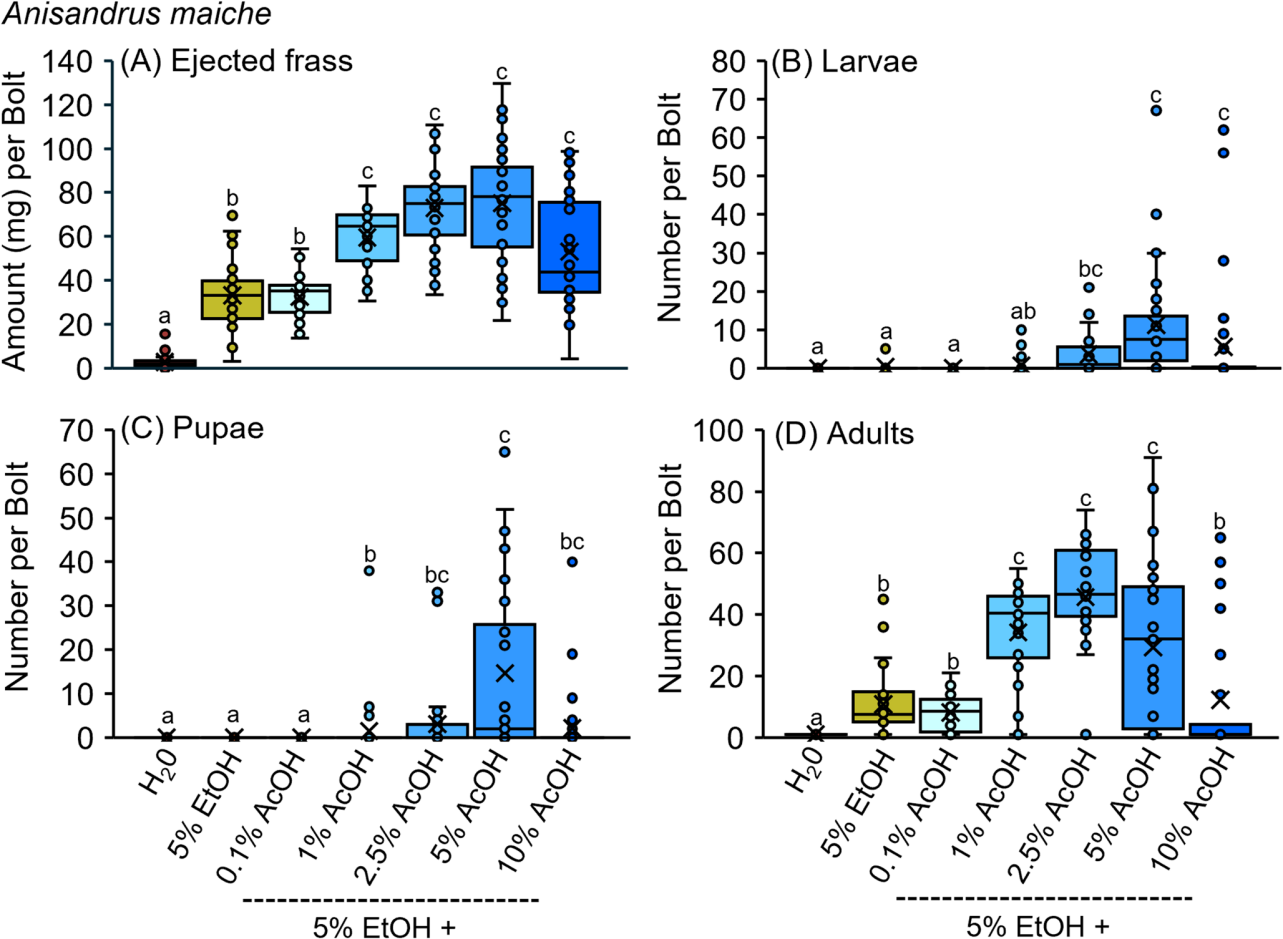
**A-D** Bolts of *A. saccharum* infused with dilutions of ethanol (EtOH) and acetic acid (AcOH) were infested with adult *A. maiche* for 35 days, then dissected and quantified for (**A**) ejected frass, (**B**) eggs, (**C**) larvae, and (**D**) pupae. Different letters above bars within each figure represent significantly different means (GLM with Tukey’s HSD: **A***Χ*^*2*^ = 329.78; df = 6; *P* < 0.0001; **B***Χ*^*2*^ = 89.55; df = 6; *P* < 0.0001; **C***Χ*^*2*^ = 61.31; df = 6; *P* < 0.0001; **D** *Χ*^*2*^ = 171.67; df = 6; *P* < 0.0001; *n* = 36 bolts per treatment)

The chemical state within host tissues increased *A. maiche* offspring production and gallery excavation. The tunneling activity and offspring production of *A. maiche* were measured after beetles were confined to *A. saccharum* bolts infused with water, 5% ethanol, and mixtures of 5% ethanol plus 0.1%, 1%, 2.5%, 5%, and 10% acetic acid (Figs. 1E-H and 5A-D). At 35 days after infesting, significantly more frass was ejected by beetles confined to bolts infused with the mixtures of 5% ethanol plus 1%, 2.5%, 5%, and 10% acetic acid than from any of the other treatments (Fig. 5A). Beetles confined to bolts infused with 5% ethanol and 5% ethanol plus 0.1% acetic acid ejected more frass than the water control. Significantly less frass was ejected from the water control than from any other treatment.

Significantly more larvae of *A. maiche* were collected from bolts infused with 5% ethanol plus 5% acetic acid and 5% ethanol plus 10% acetic acid than from all other treatments except for 5% ethanol plus 2.5% acetic acid. Similarly, more larvae of *A. maiche* were collected from bolts infused with 5% ethanol plus 2.5% acetic acid compared to 5% ethanol plus 0.1% acetic acid, 5% ethanol alone, and the water control (Fig. 5B). Significantly more pupae were collected from bolts infused with 5% ethanol plus 5% acetic acid than from all other treatments except mixtures of 5% ethanol plus 2.5% or 10% acetic acid (Fig. 5C). Significantly more *A. maiche* adults were collected from bolts infused with 5% ethanol plus 1%, 2.5%, or 5% acetic acid than all of the remaining treatments (Fig. 5D). These treatments produced increases in *A. maiche* adults of 217.2% (~ 3.2-fold), 322.6% (~ 4.2-fold), and 171.2% (~ 2.7-fold) for bolts infused with 5% ethanol plus 1%, 2.5%, and 5% acetic acid compared to 5% ethanol alone, respectively. More adults were also recovered from bolts infused with 5% ethanol plus 0.1% or 10% acetic acid, and 5% ethanol alone, than the water control (Fig. 5D).

### X-Ray Micro-CT confirms Larval-driven Gallery Expansion

**Fig. 6:**
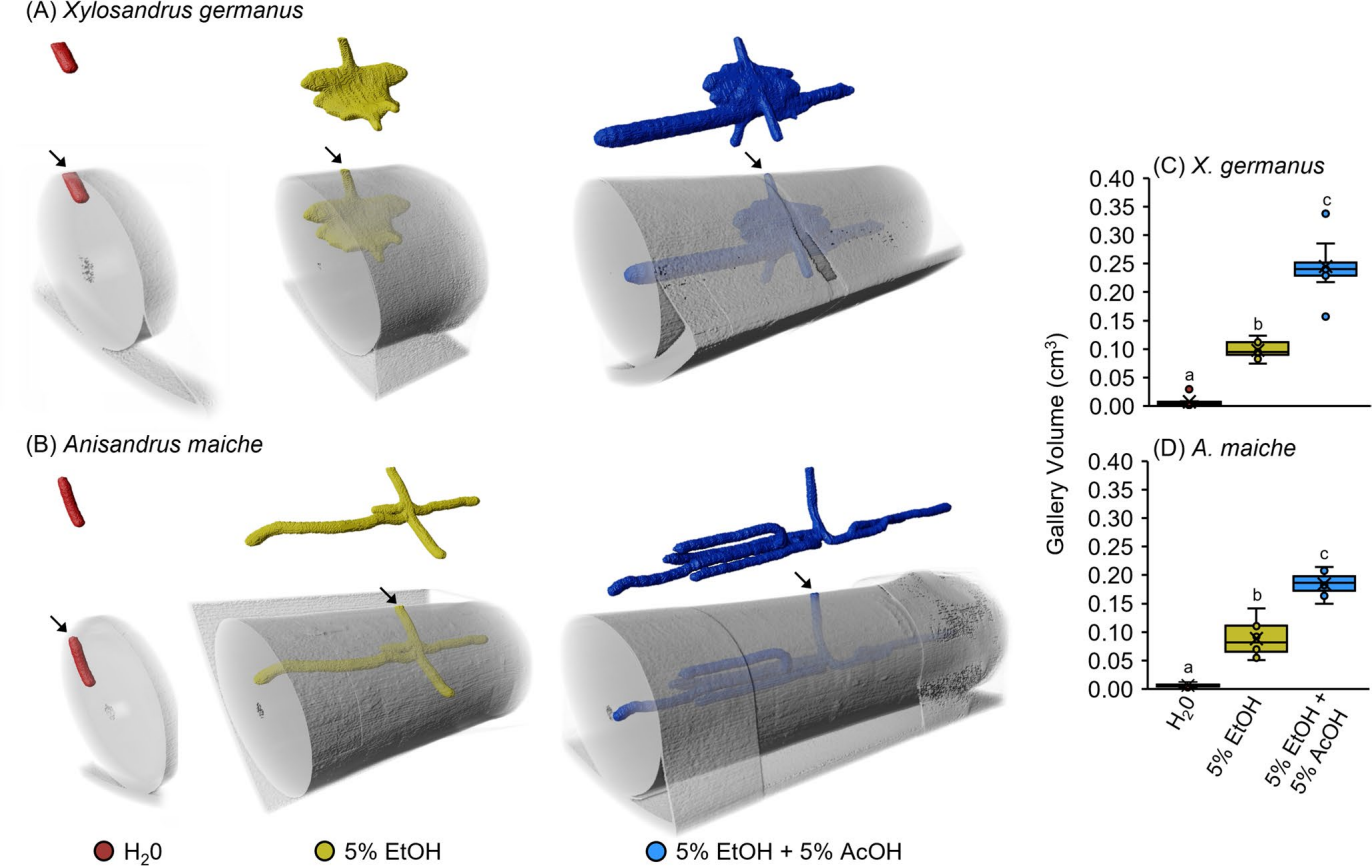
**A-D** Representative images at 21 days of galleries created by (**A**) *X. germanus* in *C. florida* bolts and (**B**) *A. maiche* in *A. saccharum* bolts infused with water, 5% ethanol (EtOH), and a mixture of 5% ethanol and 5% acetic acid (AcOH). Corresponding box plots of gallery volumes created by (**C**) *X. germanus* and (**D**) *A. maiche* in bolts. Different letters indicate significantly different means within each figure (GLM with Tukey’s HSD: **C***Χ*^2^ = 49.88; df = 2; *P* < 0.0001; *n* = 9 bolts per treatment; **D** *Χ*^2^ = 58.65; df = 2; *P* < 0.0001; *n* = 7–8 bolts per treatment)

Micro-CT scans revealed the galleries of *X. germanus* and *A. maiche* (Fig. S1). At 21 days after infesting with *X. germanus*, micro-CT scanning revealed the 3-D structure of galleries created in *C. florida* bolts infused with water, 5% ethanol, and a mixture of 5% ethanol plus 5% acetic acid (Fig. 6A). The tunnels of *X. germanus* found in bolts infused with water were superficial and unbranched. In contrast, bolts infused with 5% ethanol contained expansive, “cave-like” galleries. Tunneling by *X. germanus* expanded beyond the “cave-like” gallery into the pith when acetic acid was present. Gallery volume in bolts infused with a mixture of 5% ethanol plus 5% acetic acid was significantly higher than in bolts treated with just 5% ethanol and the water control (Fig. 6C). Additionally, a larger gallery volume was measured within bolts infused with 5% ethanol compared to the water control.

At 21 days after infesting with *A. maiche*, micro-CT scanning of *A. saccharum* bolts infused with just water revealed simple and unbranched tunnels (Fig. 6B). In contrast, bolts infused with 5% ethanol or a mixture of 5% ethanol plus 5% acetic acid contained branched tunnels that reached the pith of the bolt (Fig. 6B). Extensive branched tunneling by *A. maiche* occurred in bolts infused with a mixture of 5% ethanol plus 5% acetic acid. The gallery volume in bolts infused with a mixture of 5% ethanol plus 5% acetic acid was significantly higher than in bolts treated with 5% ethanol alone and the water control (Fig. 6D). Bolts infused with 5% ethanol also contained a larger gallery volume than the water control.

**Fig. 7:**
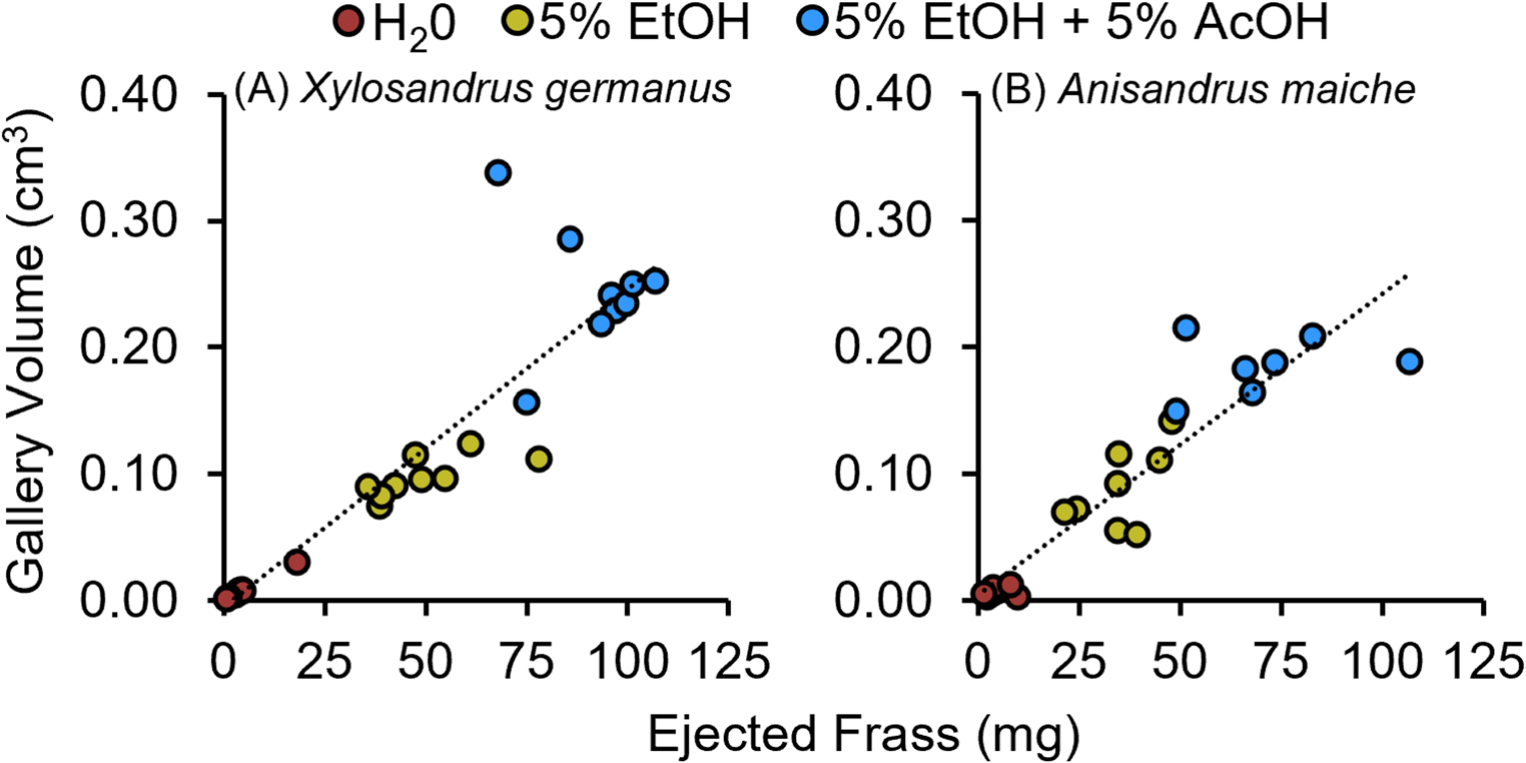
**A-B** Relationship between ejected frass and gallery volume created by (**A**) *X. germanus* and (**B**) *A. maiche* in bolts soaked in water, 5% ethanol (EtOH), and a mixture of 5% ethanol and 5% acetic acid (AcOH) (Spearman’s rank correlation: **A***r*_*s*_ = 0.95; *P* < 0.0001; *n* = 27 bolts; **B** *r*_*s*_ = 0.93; *P* < 0.0001; *n* = 23 bolts)

Spearman’s rank correlation revealed a significant positive association between ejected frass, a proxy for excavation labor, and gallery volume created by *X. germanus* within *C. florida* and *A. maiche* within *A. saccharum* bolts across all treatments: water, 5% ethanol, and a mixture of 5% ethanol plus 5% acetic acid (Fig. 7A-B). Because gallery volumes were quantified before the offspring of *X. germanus* and *A. maiche* matured into adults, the significant increase in volume confirms that the ethanol-acetic acid mixture increases gallery excavation by larvae.

### Metabolites Stimulate the Growth of the Nutritional Fungal Mutualist

The relative abundance of *A. grosmanniae*, the fungal mutualist of *X. germanus*, was determined using qPCR from galleries formed in *C. florida* bolts infused with water, 5% ethanol alone, and 0.1%, 1%, 5%, and 10% acetic acid alone (Fig. S2A). The relative amount of *A. grosmanniae* was significantly higher in bolts infused with 5% and 10% acetic acid than all other treatments, except for 5% ethanol (Fig. S2A). The relative amount of *A. grosmanniae* was also significantly higher in bolts infused with 5% ethanol than in those infused with 0.1% acetic acid and the water control.

An agar plate bioassay examined the effect of acetic acid on *A. grosmanniae* (Fig. S2B). The dry weight of *A. grosmanniae* was significantly higher when grown on media with 0.005% (v/v) acetic acid compared to those with 0.0005% acetic acid and the water control (Fig. S2B). Additionally, the dry weight of *A. grosmanniae* was higher when grown on media with 0.0005% than the water control.

### Infused Bolts Mimic the Chemical Profile of Flood-Stressed Host Trees

The concentration of acetic acid in a mixture of cambium, sapwood, and heartwood tissue samples from flood-stressed *C. florida* trees was statistically similar to that of bolts infused with a mixture of 5% ethanol plus 5% acetic acid (Table S1). The ethanol concentration in stem tissue samples from flood-stressed *C. florida* was not significantly different from that detected in bolts infused with 5% ethanol alone or in a mixture of 5% ethanol plus 0.1%, 1%, 5%, and 10% acetic acid (Table S1). Acetic acid and ethanol were not detected in the stems of non-flooded *C. florida* trees, nor in the water-infused bolts.

## Discussion

The preference-performance (“mother-knows-best”) hypothesis suggests that insects select hosts that are best suited for their offspring to increase the likelihood of successful development (Gripenberg et al. 2010). This concept explains the utilization of ethanol-enriched trees by ambrosia beetles (Ranger et al. 2018; Lehenberger et al. 2021a). We demonstrate that the combination of ethanol and acetic acid promotes offspring production and gallery development by *X. germanus* and *A. maiche*, thereby leading to a higher incidence of adult emergence for both invasive species. While the foundress initiates gallery excavation and colony establishment before transitioning to guarding the entrance (Biedermann and Taborsky 2011; Nuotclà et al. 2014; Milbrath et al. 2024), the presence of ethanol and acetic acid stimulates larval expansion of the gallery system. Behavioral observations of their feeding habits (Roeper 1995) as well as enzymes found in frass and on gallery walls indicate that adults and larvae occupy distinct feeding guilds (De Fine Licht and Biedermann 2012), highlighting differences in behavioral contributions and division of labor (Biedermann and Taborsky 2011). Ethanol and acetic acid not only select the fungal mutualist through a biological screening mechanism (Archetti et al. 2011; Ranger et al. 2018) but, as we show here, also elicit crucial digging labor by the larvae, which is necessary for rapid colony development and increased adult emergence. More generally, our results demonstrate how fungus-farming ambrosia beetles have evolved to take advantage of stress-induced metabolites for their fitness benefit; in this case, using the metabolites ethanol and acetic acid to facilitate the growth and increased production of their diet, a specialized fungal mutualist.

Gallery architecture of ambrosia beetles is an indicator of host quality, with superficial, unbranched tunnels indicating an unsuitable substrate and branched tunnels indicating the initiation of active farming (Cambronero-Heinrichs et al. 2024, 2025). In a previous study, micro-CT scanning demonstrated that tree genera and flood stress both influence the occurrence of branched tunnels made by *Anisandrus dispar* (Fabricius), *Xyleborinus saxesenii* (Ratzeburg), *X. crassiusculus*, and *X. germanus* (Cambronero-Heinrichs et al. 2024). Branching usually occurs when the foundress determines that the substrate is suitable for cultivating their nutritional fungus, and this is when eggs are laid (Cambronero-Heinrichs et al. 2024). In our study, galleries in water-infused bolts remained superficial and unbranched, confirming that the foundress assessed the substrate as poorly suitable. In contrast, the presence of ethanol increased branching, and this effect was significantly amplified by the addition of acetic acid. Notably, the expansive, ‘cave-like’ galleries (Kabe 1955) characteristic of well-established *X. germanus* colonies were primarily observed in the 5% ethanol plus 5% acetic acid treatment. These structural changes in the gallery’s complexity provide physical evidence that host metabolites serve as key signals for both colony establishment and the subsequent expansion of the gallery system.

Bolts infused with acetic acid and ethanol led to increased production of offspring from both *X. germanus* and *A. maiche*. Specifically, the addition of acetic acid resulted in adult emergence increases of ~ 3.5-fold for *X. germanus* and 2.7-fold to 4.2-fold for *A. maiche* compared to ethanol alone. The magnitude of this effect suggests that localized increases in ethanol and acetic acid could substantially increase beetle populations under natural conditions by enhancing the reproductive output per gallery. This reproductive success is likely driven by a combination of increased farming surface area and the optimized abundance and nutritional quality of the fungal mutualist. Ambrosia fungi absorb and assimilate essential elements from the surrounding xylem into their fruiting structures (i.e., conidiophores and conidia), making these nutrients available to their beetle hosts as food (Lehenberger et al. 2021b). Two interchangeable growth types of the mutualist fungi have been described: a filamentous hyphal form and a yeast-like form with conidia (Roeper 1972). The availability of the nutritionally superior yeast-like form relative to filamentous hyphae is an important bottleneck to overcome for proper larval development (French 1972; French and Roeper 1972; Secchi and Zwieniecki 2016). The yeast-like form is nutritionally vital and grows in beetle-occupied galleries (French 1972; French and Roeper 1972). In contrast, the filamentous hyphae may not provide the same quality of nutrition as shown with the ambrosia beetle *Anisandrus dispar* F., which could not complete its development when it fed solely on the filamentous hyphae (French 1972; French and Roeper 1972). Further research will be necessary to characterize the nutritional quality of ambrosia beetle fungal mutualists in the presence of acetic acid, ethanol, and other stress-induced metabolites.

Fungal mutualists in various ambrosia beetle systems exhibit optimal growth and conidiogenesis under acidic conditions, typically at pH 5.1–6.5, but they fail to thrive in more alkaline environments (Roeper 1972; French 1972; French and Roeper 1972; Saucedo-Carabez et al. 2018; Zhou et al. 2018; Joseph et al. 2021). Previous studies have shown that water stress naturally lowers xylem pH in trees (Secchi and Zwieniecki 2016), suggesting that beetles and their fungi have evolved to exploit the specific chemical environment of a weakened tree. Our results suggest that acetic acid may facilitate this morphological transition by acidifying the substrate, thereby providing a nutritional benefit of the mutualist that enhances oviposition and colony fitness. Acetic acid could also have activated fungal enzymes to oxidize lignin, facilitating the degradation of woody tissue and making nutrients more readily available to the fungus and offspring (Dashtban et al. 2010). Ethanol and acetate can also serve as direct carbon sources for the mutualistic fungi, thereby providing a metabolic advantage; however, metabolizing acetate, the conjugate base of acetic acid, would likely be more efficient (Shepardson 2013). These compounds can also act as selective antimicrobial agents that inhibit the growth of competing ‘weed’ fungi while benefitting the fungal mutualist (Ranger et al. 2018; Lehenberger et al. 2021a, b). Additional studies are warranted to explore these potential mechanisms to understand how host tree chemistry benefits the fungal mutualist and subsequent colonization by ambrosia beetles.

The mechanism by which ethanol and acetic acid facilitate gallery expansion by the larvae remains to be elucidated. As ambrosia beetle larvae ingest their symbiotic fungus and fungus-infected wood to acquire nutrients (De Fine Licht and Biedermann 2012), the synergy between acetic acid and ethanol may act as feeding stimulants, enabling larvae to expand the gallery and thereby create physical space essential for ongoing fungal cultivation and enhancing colony fitness. Ethanol and acetic acid are key stimulants in other systems, including fruit flies (*Drosophila* spp.) that feed on fresh or decaying fruit (Keesey et al. 2015, 2025; Devineni et al. 2019; Joseph et al. 2009; Kim et al. 2018; Azanchi et al. 2013). Acetic acid stimulates gustatory neurons in starved *Drosophila melanogaster* Meigen, eliciting an appetitive response (Devineni et al. 2019). These neurons also guide the strong preference of *D. melanogaster* to lay eggs in the presence of acetic acid (Joseph et al. 2009; Kim et al. 2018; Azanchi et al. 2013), and larvae and pupae that develop from eggs laid on media infused with acetic acid and ethanol exhibit higher fitness (Azanchi et al. 2013). Our results establish a chemical framework for understanding host-mediated fungal morphogenesis, providing a pathway to investigate how tree stress influences the nutritional dynamics of ambrosia beetle-fungal mutualisms. One consideration for future studies is to drench roots with acetic acid and ethanol (Ranger et al. 2018) solutions to more closely mimic a flood-stressed tree. However, the amount of acetic acid absorbed by the roots and transported throughout plants is low, whereas ethanol is readily absorbed (Corseuil et al. 2001, Kim et al. 2017).

## Conclusions

Our study provides novel insight into how acetic acid enhances tunneling and brood production.

within tree stems by two ambrosia beetles, *X. germanus* and *A. maiche*. While these stress-induced metabolites are typically toxic by-products of fermentative metabolism, ambrosia beetles have adapted them into essential chemical cues (Ranger et al. 2018, 2021; Lehenberger et al. 2021a; Cavaletto et al. 2023; Reding et al. 2025) (Fig. S3). Foundresses of both *X. germanus* and *A. maiche* opportunistically infest trees that are respiring anaerobically to create galleries for cultivating their nutritional fungal mutualists and rearing their offspring. Ethanol and acetic acid signal host-seeking ambrosia beetles, prompting tunneling behavior in the foundress once a vulnerable host is located. During gallery establishment, substrates enriched with ethanol and acetic acid can promote the growth of the yeast-like ambrosia fungus (Roeper 1972; French 1972; French and Roeper 1972), which conceivably leads to increased egg-laying and improved offspring production. Critically, since larvae obtain nutrients by consuming both the fungal gardens and the surrounding fungus-infected wood (De Fine Licht and Biedermann 2012), the combination of ethanol and acetic acid may act as feeding stimulants that promote active gallery expansion. This division of labor would allow the female to block the tunnel entrance (Nuotclà et al. 2014; Milbrath et al. 2024) while gallery expansion continues to facilitate increased farming and food production. This coordination not only facilitates increased food production but may also increase the likelihood of outbreeding and symbiont exchange (Scabbio et al. 2026). Ultimately, plant chemistry creates a physiological environment that promotes fungal productivity and facilitates larval investment in labor.

## Supplementary Information

Below is the link to the electronic supplementary material.

Supplementary Material 1 (DOCX 850 KB)

## Data Availability

The datasets supporting this article are available through an Ag Data Commons repository. https://agdatacommons.nal.usda.gov/articles/dataset/Ranger_et_al_2026_J_Chem_Ecol_Submission_ID_1ef16994-4dac-40e6-b7f3-f475afd77afd/31434886.
