# Supplementary material for: Synergistic Effects of Acetic Acid and Ethanol on Offspring Production and Gallery Expansion by Fungus-Farming Ambrosia Beetles
Source: J Chem Ecol. 2026 May 30;52(3):50. doi: 10.1007/s10886-026-01725-3 (PMC13222262; doi:10.1007/s10886-026-01725-3)
Supplement: Supplementary file 1 — Supplementary Material 1 (DOCX 850 KB) [file 10886_2026_1725_MOESM1_ESM.docx]

**Supporting Information: SI Text: Methods and SI Results: Figures S1 to S4 and Table S1**

**SI Text: Methods**

**Molecular Quantification of *A. grosmanniae* Titer.** To determine the relative abundance of the *A. grosmanniae* within galleries, we infused *C. florida* bolts with water, 5% ethanol, or acetic acid at 0.1%, 1%, 5%, and 10% (v/v) and infested them with *X. germanus*. After 21 days, we harvested and ground the gallery tissues in liquid nitrogen. We isolated total DNA using a FastDNA Kit (MP Biomedicals, CA, USA). We performed PCR using the isolated DNA showing a 260/280 ratio of 1.8 – 2. The 25 µl of reaction mixture contained 1X GoTaq Colorless Master Mix (Promega, Madison, USA), 25 µM forward and reverse primers, and 150 ng of DNA. PCR parameters were as follows, initial denaturation at 95 °C for 2 min followed by 94 °C for 45 sec, 53 °C for 45 sec and 72 °C for 2 min for 35 cycles. The final extension was at 72 °C for 7 min. Specificity of primers and the amplicon size of ITS region of *A. grosmanniae* and 28S rRNA of *C. florida* was confirmed on 1% agarose gel (Fig. S4). Additionally, we quantified *A. grosmanniae* DNA via qPCR using a CFX96 Real-Time qPCR System (Bio-Rad Laboratories, Hercules, CA, USA) and SsoFast EvaGreen Supermix. Fungal titers were normalized to the *C. florida* 28S rRNA (Accession AY967758) as an internal control. Primers targeting the fungal ITS region (Accession NR_154702) and host 28S rRNA were designed using PRIMER3. The following primers were used: ITS Forward: 5’CTTCGCTTGGTGTTGGAGGA3’, ITS Reverse: 5’CAGGGAAGCTGCTTGTACTACA3’, 28S rRNA Forward: 5’GGCCTACTATGGTGGTGACG3’, and 28S rRNA Reverse: 5’CTTGGATGTGGTAGCCGTTT3’. Each 20 µl reaction contained 25 ng of template DNA, determined to be the optimal amount via standard curve analysis (25 to 1.56 ng) to ensure high reaction efficiency and absence of host-DNA inhibition. The qPCR thermal profile consisted of 95 °C for 30 s, followed by 40 cycles of 95 °C for 10 s and 53 °C for 20 s. We performed all reactions in triplicate and confirmed target specificity via melt curve analysis and gel electrophoresis.

**Acetic Acid and Growth of *A. grosmanniae*.** We maintained *A. grosmanniae* OH11 cultures on malt extract agar (MEA; 3% malt extract, 0.5% soya peptone, w/v). To evaluate the impact of acetic acid on fungal biomass (dry weight), we amended MEA with acetic acid to achieve dilutions of 0% (water control), 0.0005%, and 0.005% (v/v) following established protocols (21).

We placed a sterile cellophane membrane (Research Products Int’l Corp, IL) over the solidified agar and inoculated the center with a 3-mm mycelial plug. Plates were photographed every 2 days, and the surface area was measured using ImageJ 1.47v (National Institutes of Health, Bethesda, MD, USA). At 6 days post-inoculation, we harvested the mycelial mats, recorded fresh weights, and dried them at 37 °C to a constant mass. The mycelial density was calculated as dry weight per unit surface area.

**Quantification of Acetic Acid and Ethanol.** We measured ethanol and acetic acid concentrations in infused *C. florida* bolts and compared them with those of experimentally flood-stressed trees. The concentrations of acetic acid and ethanol were measured in bolts treated with water, 5% ethanol, or 5% ethanol plus acetic acid at dilutions of 0.1%, 1%, 5%, or 10% (v/v). After soaking, the *C. florida* bolts were incubated at 25 °C. A subset of the bolts was removed from the incubator at 3 and 7 days, and transferred to -80 °C until further processing. For the flooded trees (*n* = 4), we induced anaerobic stress in living *C. florida* trees using a pot-in-pot system (37), with non-flooded trees as controls (*n* = 4). At 7 days after initiating flooding, we harvested the stems as bolts and stored them at -80 °C. To sample tissue from the infused bolts and flood-stressed trees, we drilled to 1.0 cm depth in the center of each bolt to collect 80 mg of shavings (cambium, sapwood, and heartwood) from each bolt. Samples were placed in 2 mL borosilicate glass vials with a 7 mm filter paper disc. We added 10 μL aliquot of 0.5 μg/mL *tert*-butanol-d_10_ (Sigma-Aldrich, St. Louis, MO) as an internal standard before sealing the vials with a screw-top cap and septum.

We equilibrated the vials in a 40 °C water bath for 5 min, then exposed a CAR/PDMS SPME fiber (carboxen/polydimethylsiloxane; 75 µm coating; Sigma-Aldrich, St. Louis, MO, USA) to the headspace for 5 min. The fiber was thermally desorbed at 250 °C for 2 min in an Agilent 7890B GC (Agilent Technologies, Palo Alto, CA, USA) with a SPME liner (0.75 mm i.d. × 6.35 mm × 78.5 mm, i.d. × o.d. × length; Restek, Bellefonte, PA, USA) under splitless mode with 2 min splitter off time. A DB-5MS column (0.25 mm × 30 m × 0.25 µm, inner diameter × length × film thickness; Agilent J&W, Santa Clara, CA, USA) was used with a temperature program of 40 °C (4 min), ramped to 200 °C at 15 °C/min. An Agilent 5977A MS (scan range of 33–120 amu) was operated in electron impact mode. Acetic acid and ethanol were identified by comparing their mass spectral fragmentation patterns and corresponding retention times with those of authentic standards, referencing the National Institute of Standards and Technology (NIST) MS database. We quantified ethanol (*m/z* 45, 46) and acetic acid (*m/z* 60) relative to the internal standard (*m/z* 65) using external standard curves.

**SI Results: Figures S1 to S4 and Table S1**


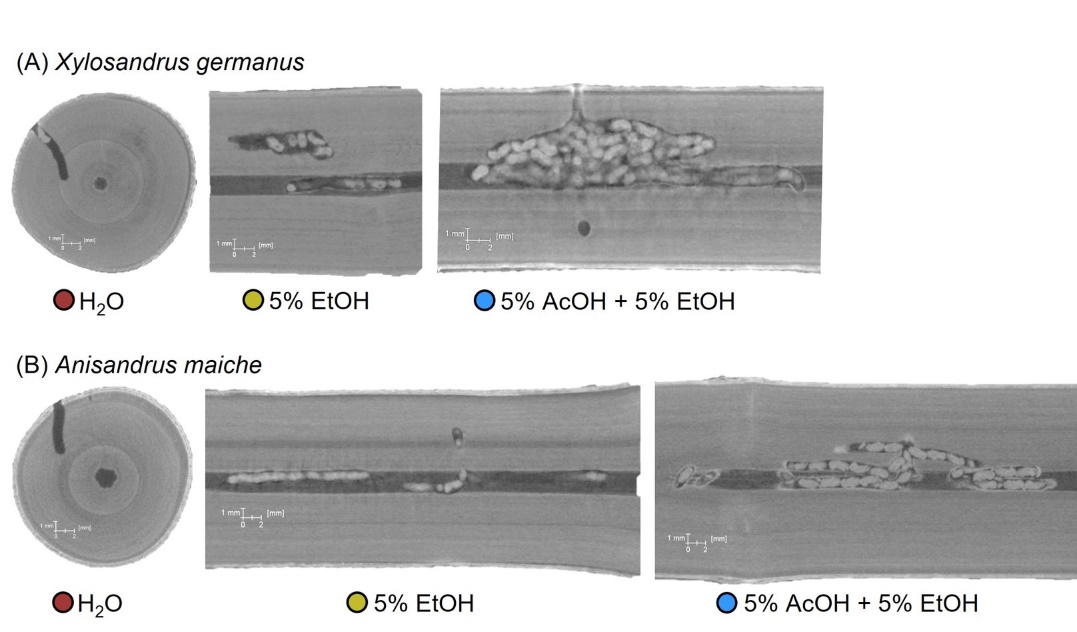


**Fig. S1A-B.** Representative X-ray micro-computed tomography (μ-CT) images taken at 21 days after confining an individual (A) *Xylosandrus germanus* to a stem section of *C. florida* and (B) *Anisandrus maiche* to *A. saccharum* infused with dilutions of water, ethanol (EtOH), and acetic acid (AcOH).


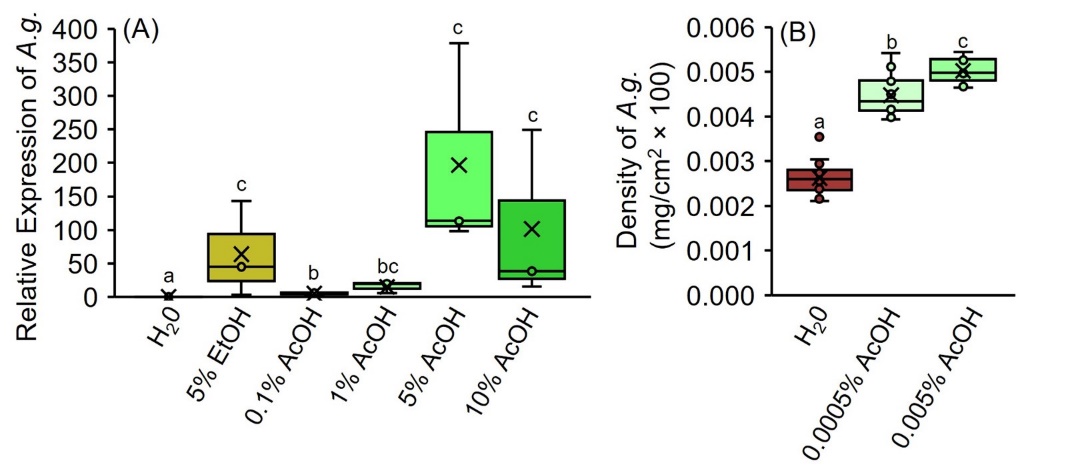


**Fig. S2A-B.** (A) Relative quantification of *Ambrosiella grosmanniae* (*A.g.*), the nutritional fungal mutualist of *Xylosandrus germanus*, was measured within galleries of *C. florida* bolts infused with dilutions of ethanol (EtOH) and acetic acid (AcOH). (B) The density of *A. grosmanniae* was measured on agar plates containing acetic acid at 0%, 0.0005%, and 0.005% (v/v). Different letters above bars represent significantly different means ((A) *Χ^2^* = 45.48; df = 5; *P* <0.0001; *n* = 3 bolts per treatment; (B) *Χ^2^* = 72.41; df = 2; *P* <0.0001; *n* = 12 plates per treatment).


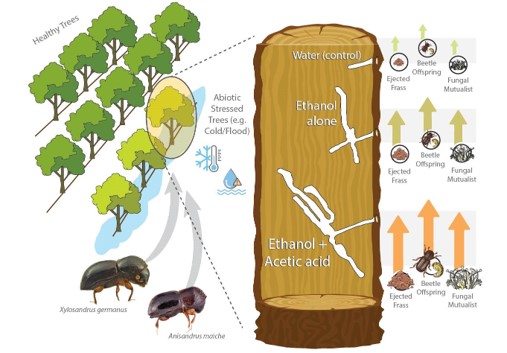


**Fig. S3.** **Conceptual model of host-metabolite-driven social coordination and fungus-farming in ambrosia beetles.** Invasive foundresses of *Xylosandrus germanus* and *Anisandrus maiche* are attracted to stressed trees (e.g., flooded or freeze-stressed) emitting ethanol and acetic acid from fermentative metabolism. These metabolites serve a dual role: (A) they act as semiochemical cues that stimulate tunneling by the foundresses, and (B) they promote the growth of the nutritionally superior yeast-like form of their fungal mutualist that serves as the sole source of food for the larvae and adults. This optimized environment stimulates enhanced oviposition by the foundress. Furthermore, because larvae obtain nutrients from both the fungal gardens and the surrounding fungus-infected wood, the synergistic effect of ethanol and acetic acid likely acts as a feeding stimulant, promoting active gallery expansion by the larvae. This division of labor enables the foundress to guard the gallery entrance for colony defense, while the larvae engineer the physical space required for expanding fungal cultivation and colony growth.


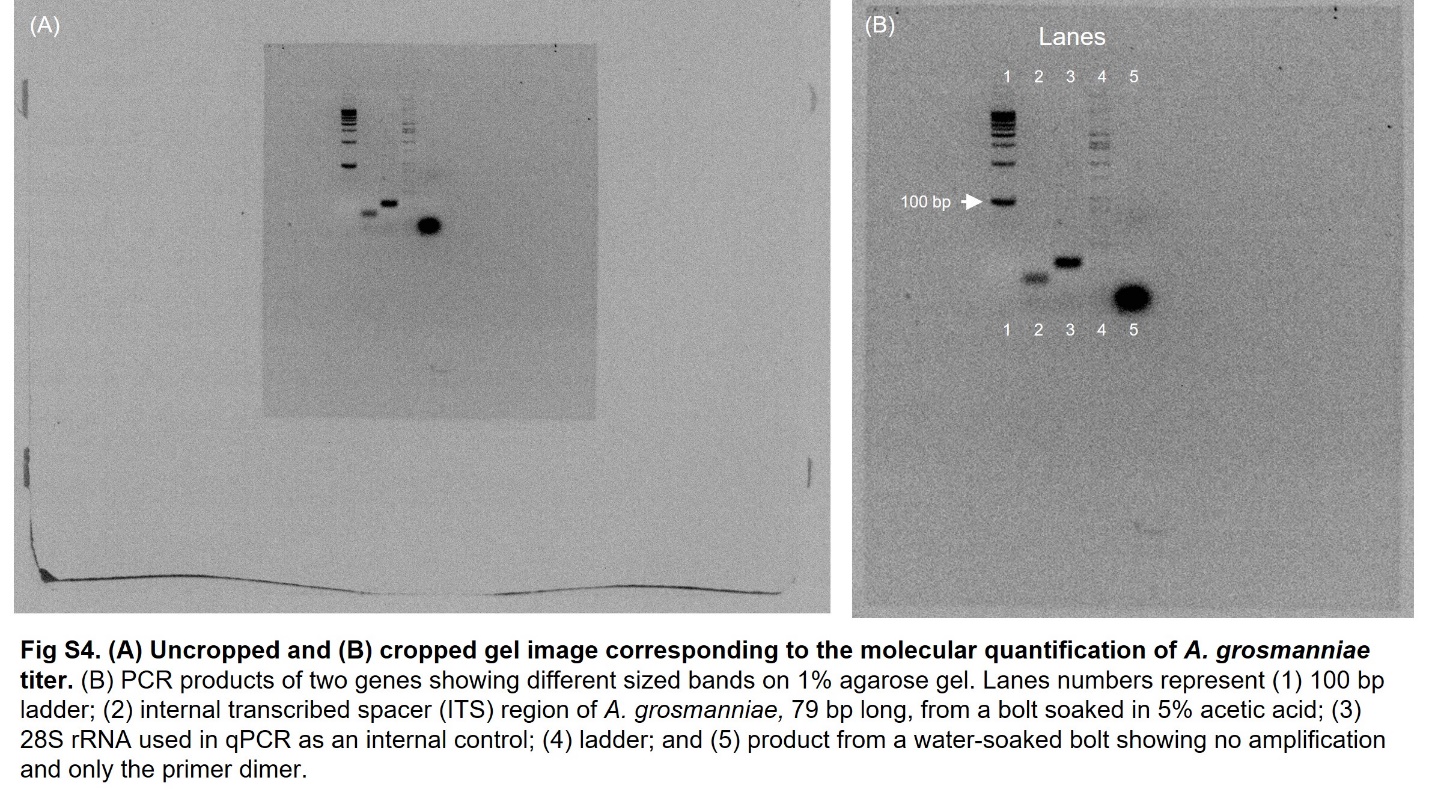


**Fig. S4. (A) Uncropped and (B) cropped gel image corresponding to the molecular quantification of *A. grosmanniae* titer.** (B) PCR products of two genes showing bands on 1% agarose gel. Lanes numbers represent (1) 100 bp ladder; (2) internal transcribed spacer (ITS) region of *A. grosmanniae,* 79 bp long, from a bolt soaked in 5% acetic acid; (3) 28S rRNA, 83 bp long, from *C. florida* bolt soaked in 5% acetic acid as an internal control during qPCR; (4) DNA ladder; and (5) water-soaked bolt showing only the primer dimer and no amplification of ITS region of *A. grosmanniae.*

| **Table S1.** Quantification by SPME-GC-MS of acetic acid and ethanol content in the stems of flowering dogwood (*Cornus florida* L.) subjected to flood stress versus stem sections (i.e., bolts) infused with ethanol (EtOH) and acetic acid (AcOH). | | | | | |
| --- | --- | --- | --- | --- | --- |
| Treatment | Acetic Acid  (ng per mg of tissue) | |  | Ethanol  (ng per mg of tissue) | |
| Control Tree | 0.00 | ± 0.00a |  | 0.00 | ± 0.01a |
| Flooded Tree | 177.44 | ± 18.22c |  | 258.32 | ± 45.21b |
| H_2_0 Soaked Bolt | 0.00 | ± 0.00a |  | 0.00 | ± 0.04a |
| 5% EtOH Soaked Bolt | 27.77 | ± 2.60b |  | 251.94 | ± 17.87b |
| 5% EtOH + 0.1% AcOH Soaked Bolt | 27.42 | ± 2.69b |  | 290.30 | ± 16.69b |
| 5% EtOH + 1% AcOH Soaked Bolt | 22.33 | ± 0.77b |  | 273.10 | ± 48.93b |
| 5% EtOH + 5% AcOH Soaked Bolt | 133.45 | ± 29.28c |  | 281.86 | ± 23.23b |
| 5% EtOH + 10% AcOH Soaked Bolt | 3857.90 | ± 149.68d |  | 313.87 | ± 18.39b |
| Statistics (Χ^2^; df; *P*)^a^ | 189.44; 7; <0.0001 | |  | 148.96; 7; <0.0001 | |
| ^a^Different letters within a column indicate significantly different means (*n* = 4 bolts per treatment). | | | | | |
